# Psychometric Properties of Multiple-Choice Questions Used in Undergraduate Medical Education in India: A Systematic Review and Meta-Analysis

**DOI:** 10.7759/cureus.99299

**Published:** 2025-12-15

**Authors:** Himel Mondal, Amita Kumari, Ankita Priya, Anup Kumar Dhanvijay, Rintu K Gayen, Shaikat Mondal, Pradosh Kumar Sarangi

**Affiliations:** 1 Physiology, All India Institute of Medical Sciences, Deoghar, Deoghar, IND; 2 Electronics and Communication Engineering, Institute of Engineering and Management, Kolkata, IND; 3 Physiology, Raiganj Government Medical College and Hospital, Raiganj, IND; 4 Radiodiagnosis, All India Institute of Medical Sciences, Deoghar, Deoghar, IND

**Keywords:** benchmarking, item analysis, mcq, medical education, multiple-choice questions, psychometrics

## Abstract

Multiple-choice questions (MCQs) are widely used in Indian undergraduate medical education to assess learning. High-quality MCQs are essential for reliable and valid evaluation, yet real-world psychometric performance across Indian institutions remains unclear. This study aimed to systematically review and analyze the psychometric properties of MCQs used in the assessment of undergraduate medical students to establish normative benchmarks. A systematic search of PubMed, Scopus, Web of Science, and Cochrane Central was conducted to identify studies reporting psychometric indices of MCQs in Indian undergraduate medical education. Eligible studies included those reporting difficulty index, discrimination index, distractor efficiency, internal consistency, or point biserial correlation. Data were extracted independently by reviewers and pooled using random-effects meta-analysis. Heterogeneity and publication bias were assessed. Fifteen studies with 17 datasets were included, covering multiple medical subjects and institutions. The pooled mean difficulty index (dataset = 15) was 51.5 (95% CI: 47.3-55.7%), the discrimination index (dataset = 14) was 0.29 (95% CI: 0.25-0.34), and distractor efficiency (dataset = 12) was 61.7% (95% CI: 45.5-77.9%). There was high heterogeneity between studies. However, no significant publication bias was detected for any psychometric parameter. This study provides the first comprehensive synthesis of MCQ performance in Indian medical education, highlighting moderate difficulty, generally acceptable discrimination, and wide variability in distractor effectiveness. These findings offer empirical evidence to establish normative benchmarks for supporting policy-making to enhance the quality of MCQ, aligned with the national average.

## Introduction and background

Multiple-choice questions (MCQs) are widely used in undergraduate medical education in India due to their ability to efficiently assess a broad range of knowledge areas in an objective and scalable manner [1]. High-quality MCQs are essential for ensuring the validity and reliability of assessments [2]. Psychometric properties such as difficulty index, discrimination index, distractor efficiency, and internal consistency are critical metrics for evaluating the quality of MCQs [3].

While guidelines and expert recommendations propose ideal benchmarks for these psychometric indices [4], the real-world scenario may differ significantly due to variability in item-writing practices, institutional resources, and examination conditions [5]. Existing studies in the Indian context are scattered, often methodologically inconsistent, and primarily focus on individual institutions or small sample sizes without providing a broader national perspective. Consequently, it remains unclear what the actual average values of key psychometric properties are across Indian medical institutions.

This systematic review and meta-analysis aim to fill that knowledge gap by systematically identifying, synthesizing, and deriving pooled estimates of the psychometric properties of MCQs used in undergraduate medical assessments in India. By establishing pooled national averages and highlighting the existing variability, this study would provide critical insights for educators and policymakers.

## Review

Study design

This study is a systematic review designed to synthesize the psychometric properties of MCQs used in the assessment of undergraduate medical students in India. The protocol for this review was registered in the International Prospective Register of Systematic Reviews (PROSPERO; CRD420251083866), and the objective is to provide national pooled estimates of key psychometric indices to inform evidence-based improvements in MCQ design and assessment practices.

Inclusion criteria

Studies eligible for inclusion are those conducted in India involving undergraduate MBBS (Bachelor of Medicine and Bachelor of Surgery) medical students from any year of study and any subject, enrolled in either government or private colleges, where MCQs with four response options were used as the assessment tool. Only studies published in English from the year 2000 onwards and reporting at least one psychometric property, such as difficulty index, discrimination index, distractor efficiency, internal consistency reliability measured by Kuder-Richardson Formula 20 (KR-20) or Cronbach’s alpha, or point biserial correlation, with extractable quantitative data were considered. Studies were excluded if they involved non-MBBS or postgraduate students, used non-MCQ formats or simulated questions without real student responses, did not report psychometric data, or were published as reviews, editorials, or opinion pieces. Duplicate studies and those without available full texts were also excluded.

Information sources and search strategy

A search was conducted in PubMed, Scopus, Web of Science (WOS), and Cochrane Central databases. No restriction on the publication date was applied. Although our aim was to include studies (randomized controlled trials, non-randomized controlled trials, and observational studies) after 2000, we did not restrict the search by year to avoid any exclusion of studies due to any errors in bibliographic data in the database. No country or language filter was applied, but it was later screened by the authors. The search was conducted on 1st July 2025 on all four databases.

The literature search was conducted using PubMed, WOS, Scopus, and Cochrane Central databases. In PubMed, the specified keywords were used, while in WOS, the topic search option was applied, and in Scopus, the search was restricted to title, abstract, and keywords. The search terms included the following combination: ("item analysis" OR "difficulty index" OR "discrimination index" OR “distractor efficiency” OR "test analysis" OR "psychometric analysis") AND ("multiple choice questions" OR “multiple-choice question” OR MCQ OR "single best answer") AND ("medical education" OR "medical students" OR "medical examination" OR examination OR test OR semester OR MBBS OR medical).

Study selection

The titles and abstracts of all retrieved studies were independently screened by two reviewers (A.K. and A.P.) to assess eligibility. Any discrepancies were resolved through discussion, with arbitration by a third reviewer (H.M.) when necessary.

**Figure 1:**
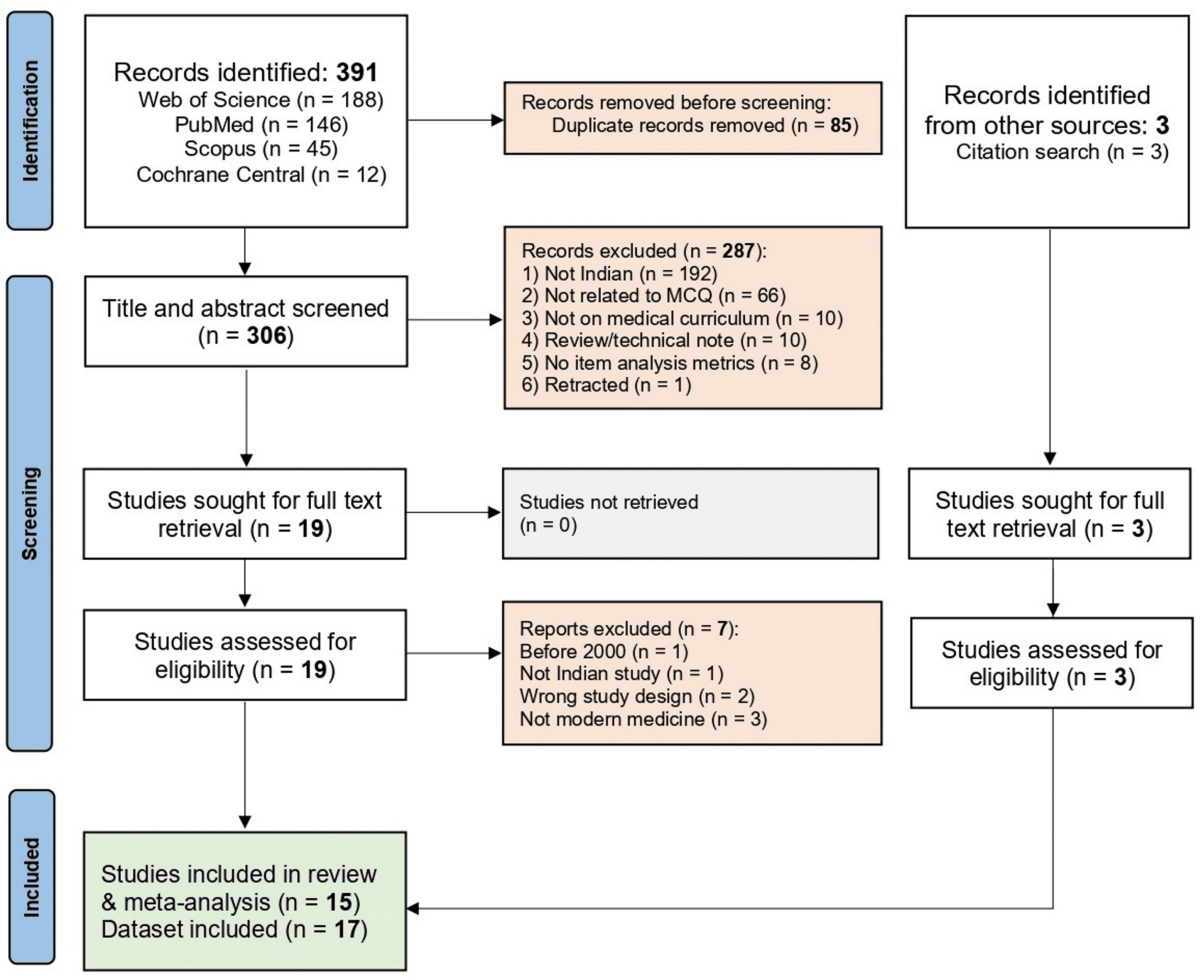
PRISMA flowchart showing studies in various phases of screening. PRISMA: Preferred Reporting Items for Systematic reviews and Meta-Analyses; MCQ: multiple-choice questions.

We identified a total of 391 articles from PubMed (n = 146), Scopus (n = 45), Web of Science (n = 188), and Cochrane Central (n = 12). After removal of duplicates (n = 86), a total of 306 articles were screened for title and abstract. A total of 287 records were excluded due to various reasons, as shown in Figure 1. After adjudication of the first (A.K.) and second (A.P.) reviewers’ selection, a total of 19 articles were sought for full-text screening.

Full-text articles of potentially eligible studies were then retrieved and assessed against the inclusion and exclusion criteria by two additional reviewers (S.M. and A.K.D.). Disagreements during full-text screening were similarly resolved through discussion, with a third reviewer serving as adjudicator.

During full-text screening, studies were excluded for the following reasons: one study was published before the year 2000 (in 1974) [6], one focused on Ayurveda, two were related to dental education, one was of an unrelated study type not addressing the medical curriculum, one was a pre-post training study of faculty but did not report relevant metrics, and one was conducted outside India. Hence, a total of 12 studies with 14 datasets were included in the study.

In addition to that, the reviewers searched for extra studies with citation scanning and found another three studies that were added to the final analysis (Figure 1). Hence, a total of 15 studies [4,7-20] were included in the systematic review, and a dataset of 17 was selected for meta-analysis.

Data extraction

Data extraction was carried out independently by two reviewers (H.M. and S.M.) in a pre-defined extraction spreadsheet (i.e., Microsoft Excel file, Microsoft Corporation, Redmond, WA). Extracted data included study characteristics such as authorship, year of publication, state where the study was conducted, sample size (number of students and number of MCQs analyzed), and reported psychometric properties.

Outcome measures

The primary outcomes of this review are the pooled estimates of key psychometric properties of MCQs used in Indian undergraduate medical assessments. These include the difficulty index, which indicates the proportion of students who answered the item correctly; the discrimination index, which reflects how well an item differentiates between high-performing and low-performing students [21]; distractor efficiency, representing the proportion of functional distractors in each item; internal consistency reliability, measured using KR-20 or Cronbach’s alpha [22]; and point biserial correlation [23], which measures the strength of correlation of the item score with the overall score.

Data synthesis and analysis

We performed a random-effects meta-analysis of continuous outcomes for the different psychometric indices. The use of a random-effects model was considered appropriate as it accounts for variability across studies. Although the included studies involved different student cohorts from various semesters and medical subjects, these differences are unlikely to substantially affect the pooled psychometric estimates because the indices themselves are statistical properties of the test items rather than characteristics of the student groups. In other words, difficulty, discrimination, distractor efficiency, reliability, and item-total correlation reflect the quality of the questions, which remains relatively stable across different cohorts. Therefore, pooling the results across diverse MBBS student groups provides a valid summary of the psychometric characteristics of MCQs used in Indian medical education.

Pooled mean estimates with 95% confidence intervals were presented, and forest plots were generated to visually display the pooled estimates and variability across studies. The heterogeneity of the included studies was assessed using the I² statistic.

Reporting

The systematic review follows the PRISMA (Preferred Reporting Items for Systematic Reviews and Meta-Analyses) guidelines to ensure transparent and complete reporting of methods, results, and conclusions.

Analysis tool

All statistical analyses and visualizations were performed using RStudio, 2024.12.1 Build 563 (Posit PBC, Boston, MA), an integrated development environment for R (R Foundation for Statistical Computing, Vienna, Austria) [24]. We performed a random-effects meta-analysis of means using the “meta” package in R. Forest plots were generated to present study-level estimates with 95% confidence intervals and the pooled mean effect. To assess publication bias, contour-enhanced funnel plots were created [25], displaying regions of statistical significance (p < 0.10, p < 0.05, and p < 0.01). Funnel plot asymmetry was further examined using Egger’s linear regression test and Begg’s rank correlation test [26]. The codes for the analysis can be obtained from the corresponding author.

Results

A total of 15 studies with 17 datasets were included in the study. The characteristics of the studies and their reported metrics are shown in Table 1. Between 2012 and 2022, studies from different states of India evaluated the psychometric properties of MCQs across various subjects, including physiology, pharmacology, pathology, ophthalmology, pediatrics, biochemistry, community and family medicine, and gynecology. The number of MCQs analyzed ranged from 20 to 240, with student sample sizes between 21 and 400.

**Table 1:** Studies and their characteristics. MCQ: multiple-choice question; P: difficulty index; D: discrimination index; DE: distractor efficiency; KR-20: Kuder–Richardson Formula 20; r_pb_: point-biserial correlation coefficient; -: data not available or obtainable.

Author	Year	State	Student number	Subject	MCQ number	Metrics reported	Difficulty	Discrimination	Distractor efficiency	Biserial	KR-20 overall
Agarwal et al. [7]	2023	Uttar Pradesh	65	Physiology	20	D	-	0.18	-	-	-
Bhat and Prasad [10]	2021	Karnataka	120	Ophthalmology	40	P, D, DE	53.22±22.44	0.26±0.16	78.32±32.11	-	-
Bhattacherjee et al. [9]	2022	West Bengal	98	Community and family medicine	60	P, D, DE	47.95±16.39	0.12±0.10	18.42±15.35	-	-
Biswas et al. [12]	2015	Madhya Pradesh	50	Biochemistry	30	P, D	39.8±14.2	0.34±0.12	-	-	-
							61.4±19.4	0.43±0.17			
Chauhan et al. [15]	2023	Gujarat	400	Multiple	200	P, D, DE	57.76±28.66	0.44±0.37	84.17±28.68	-	-
Dhanvijay et al. [16]	2023	Jharkhand	60	Physiology	100	P, D, DE	56.02±24.36	0.27±0.16	34.67±31.75	-	0.65
							57.93±21.1	0.28±0.16	45±33.96		0.87
Gajjar et al. [14]	2014	Gujarat	148	Community and family medicine	50	P, D, DE	39.4±21.4	0.14±0.19	88.6±18.6	-	-
Gupta et al. [17]	2020	Delhi	21	Multiple	100	D, P, DE	42.1±26.6	-	-	-	-
Kaur et al. [11]	2016	Punjab	150	Pharmacology	50	P, D, DE	-	-	81.98±25.41	-	-
Kulshreshtha et al. [13]	2024	Rajasthan	50	Gynecology and obstetrics	25	P, D, DE	46.3±19.4	0.3±0.1	82±19.8	-	-
Kumar et al. [4]	2021	Maharashtra	150	Physiology	90	P, D, DE, KR-20	54.95±13.4	0.31±0.12	32.35±31.3	-	0.71
Namdeo and Sahoo [18]	2016	Odisha	76	Pediatrics	25	D, P, DE	65.92±22.2	0.33±0.23	46.67±30.43	-	-
Pande et al. [19]	2013	Maharashtra	400	Physiology	240	P, D	52.53±20.59	0.3±0.18	-	-	-
Rao et al. [20]	2016	Karnataka	120	Pathology	40	P, D, DE	50.16±16.15	0.34±0.17	89.99±24.42	-	-
Vegada et al. [8]	2016	Gujarat	44	Pharmacology	30	P, D, r_pb_	48.48±19.76	0.23±0.12	1.77±0.67	0.33±0.23	-

**Figure 2:**
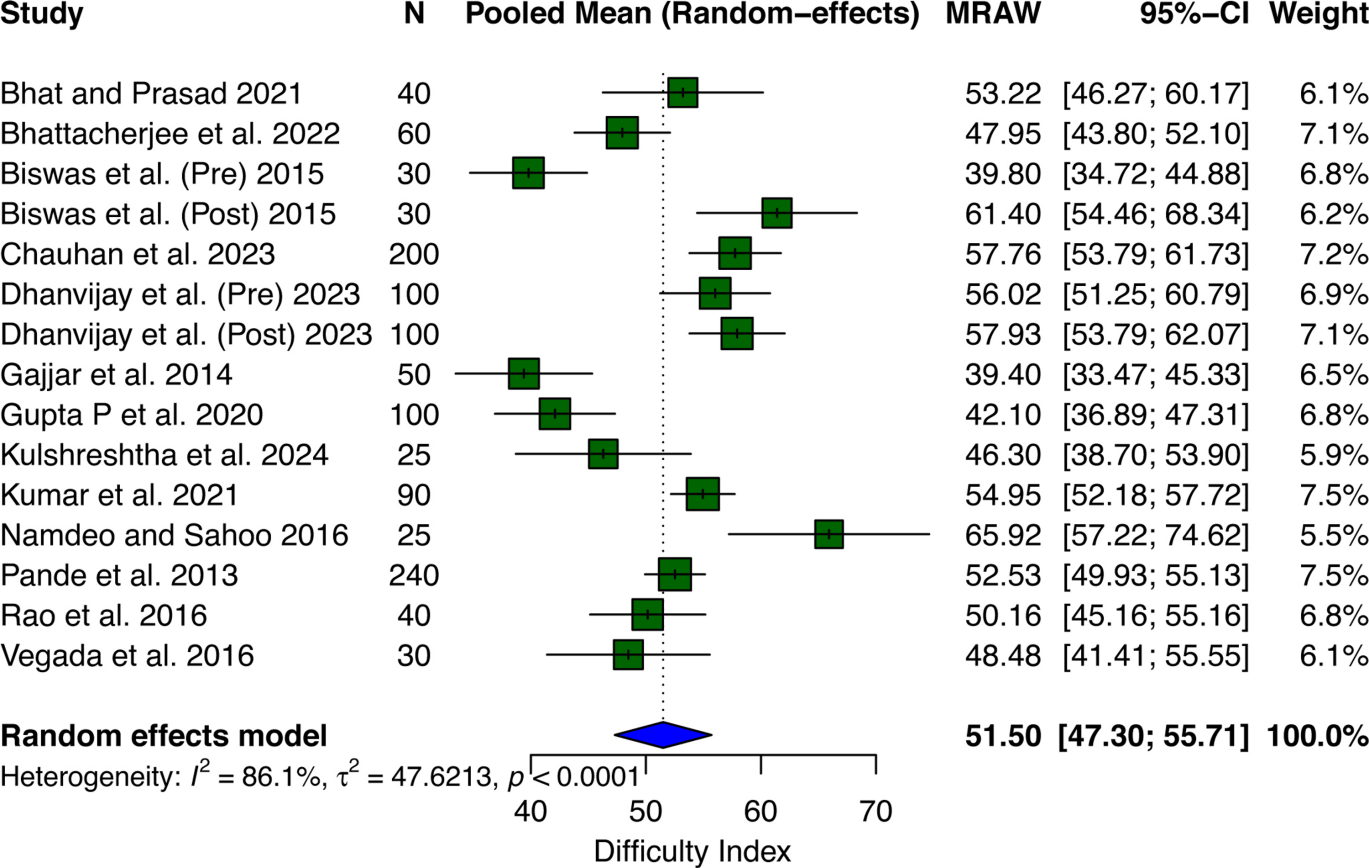
Forest plot showing the pooled mean of the difficulty index. MRAW: mean (raw scale). Bhat and Prasad [10], Bhattacherjee et al. [9], Biswas et al. [12], Chauhan et al. [15], Dhanvijay et al. [16], Gajjar et al. [14], Gupta et al. [17], Kulshreshtha et al. [13], Kumar et al. [4], Namdeo and Sahoo [18], Pande et al. [19], Rao et al. [20], and Vegada et al. [8].

Figure 2 shows the forest plot of the difficulty index among the studies. Across 15 datasets, the pooled mean difficulty index was 51.5 (95% CI: 47.30-55.71) using a random effects model. The analysis included 1,250 participants across studies conducted between 2012 and 2022, with individual study difficulty indices ranging from 39.40% to 65.92%. There was substantial heterogeneity between studies (I² = 86.1%, τ² = 47.62, p < 0.0001), indicating significant variation in difficulty levels across different contexts or populations examined.

**Figure 3:**
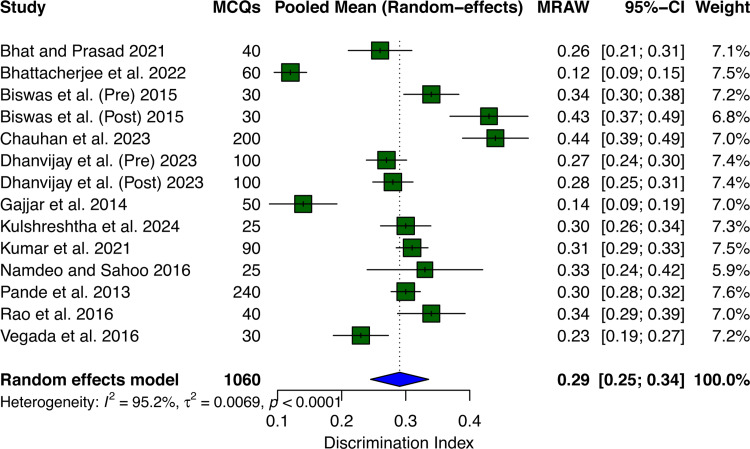
Forest plot showing the pooled mean of the discrimination index. MCQs: multiple-choice questions; MRAW: mean (raw scale). Bhat and Prasad [10], Bhattacherjee et al. [9], Biswas et al. [12], Chauhan et al. [15], Dhanvijay et al. [16], Gajjar et al. [14], Kulshreshtha et al. [13], Kumar et al. [4], Namdeo and Sahoo [18], Pande et al. [19], Rao et al. [20], and Vegada et al. [8].

Across 14 datasets involving 1,060 MCQs, the pooled mean discrimination index was 0.29 (95% CI: 0.25-0.34) using a random effects model (Figure 3). Individual study discrimination indices ranged from 0.12 to 0.44, with most studies falling within the acceptable discrimination range. However, there was very high heterogeneity between studies (I² = 95.2%, τ² = 0.007, p < 0.0001), indicating substantial variation in discrimination ability across different contexts or assessment instruments.

**Figure 4:**
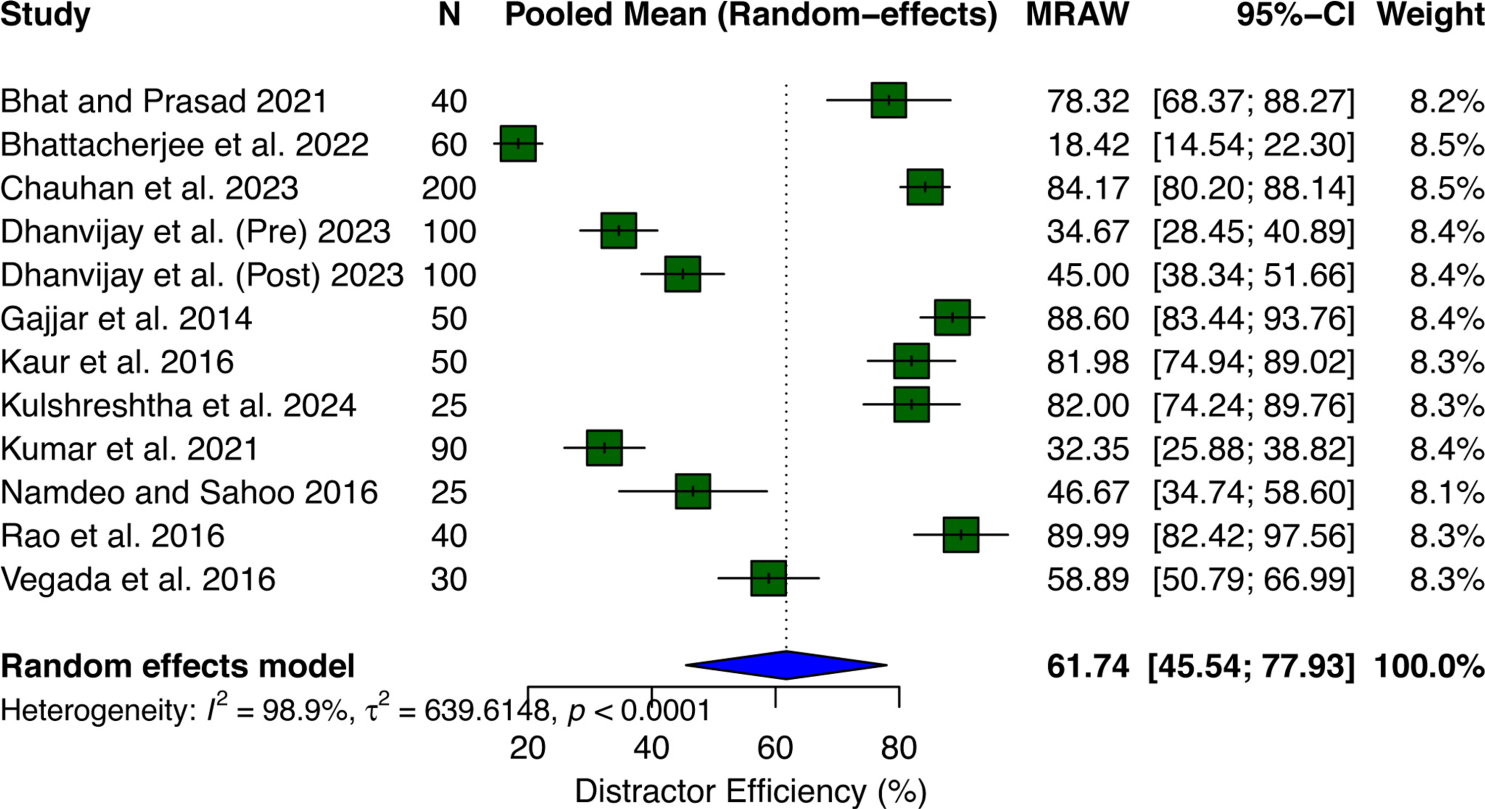
Forest plot showing the pooled mean of distractor efficiency. MRAW: mean (raw scale). Bhat and Prasad [10], Bhattacherjee et al. [9], Chauhan et al. [15], Dhanvijay et al. [16], Gajjar et al. [14], Kaur et al. [11], Kulshreshtha et al. [13], Kumar et al. [4], Namdeo and Sahoo [18], Rao et al. [20], and Vegada et al. [8].

Figure 4 shows the distractor efficiency. Across 12 datasets involving 810 participants, the pooled mean distractor efficiency was 61.74% (95% CI: 45.54-77.93%) using a random effects model. Individual study distractor efficiency values showed extreme variation, ranging from as low as 18.42% to as high as 89.99%, indicating substantial differences in the quality of distractors across different assessment contexts. There was exceptionally high heterogeneity between studies (I² = 98.9%, τ² = 639.61, p < 0.0001), representing nearly complete variability across studies.

As KR-20 was reported in two studies [4,16] and point-biserial correlation was reported in one study [8], a meta-analysis was not conducted. In addition, due to a lack of adequate data from the study by Agarwal et al. [7], it could not be included in the meta-analysis.

**Figure 5:**
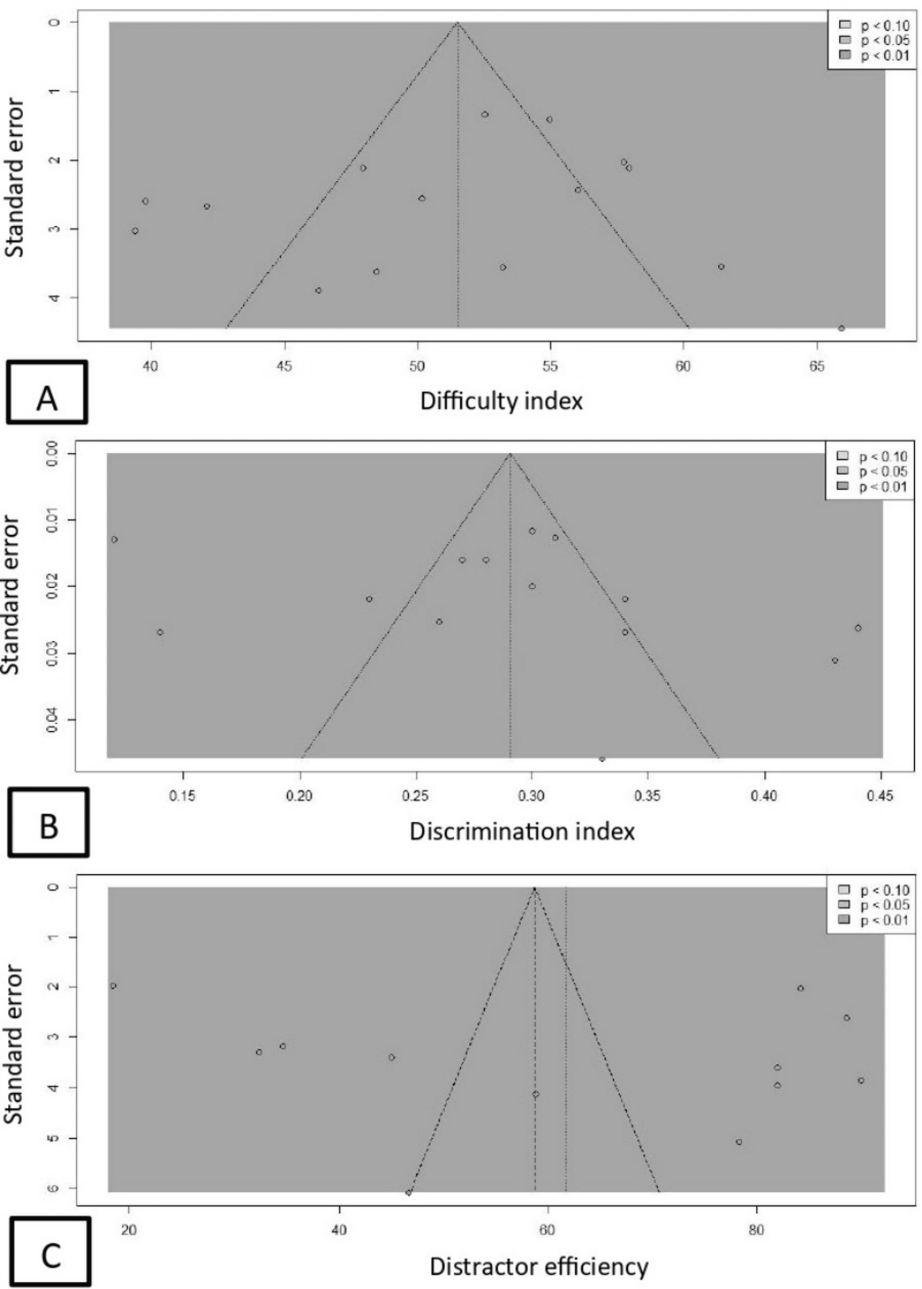
Funnel plot of three meta-analyses: (A) difficulty index, (B) discrimination index, and (C) distractor efficiency.

Publication bias was assessed using Egger's linear regression test and Begg's rank correlation test. Visual output as a funnel plot is shown in Figure 5. No test indicated significant publication bias in difficulty index (Egger's test: t = -0.66, p = 0.518; Begg's test: z = -0.74, p = 0.458) (Figure 5A), in discrimination index (Egger’s test: t = 1.09, p = 0.297; Begg’s test: z = 0.38, p = 0.701) (Figure 5B), or in distractor efficiency (Egger’s test: t = 0.58, p = 0.578; Begg’s test: z = 0.00, p = 1.000) (Figure 5C).

Discussion

This meta-analysis synthesized findings from multiple studies across different states of India, evaluating the psychometric properties of MCQs in a variety of medical subjects. The studies varied in terms of the number of MCQs analyzed and the student sample sizes, reflecting diverse educational contexts and assessment practices.

The MCQs were found to be of moderate difficulty, though there was substantial variation across studies. This suggests that the level of challenge posed by MCQs can differ depending on the subject, institutional practices, and student populations. The discrimination ability of the MCQs was generally acceptable, indicating that most items could differentiate between high- and low-performing students. However, there was considerable variation across studies, likely reflecting differences in item construction, subject complexity, or student preparedness. Distractor quality showed the greatest variability, with some items functioning well while others were less effective. Poorly performing distractors can reduce the overall reliability and validity of MCQs [27], emphasizing the importance of systematic evaluation and revision of distractors during assessment design.

From this study, the current national average difficulty index, discrimination index, and distractor efficiency have been established (Table 2). The availability of empirical data on the actual performance of MCQs in Indian medical education provides a vital foundation for educators, curriculum designers, and assessment authorities [28]. Stakeholders can compare the quality of the MCQs with the national average benchmark.

**Table 2:** Pooled mean indices and their 95% confidence intervals.

Parameter	Pooled mean	95% confidence interval
Difficulty index	51.5	47.3-55.7
Discrimination index	0.29	0.25-0.34
Distractor efficiency	61.74%	45.54-77.93%

We found only a few studies reported the KR-20, and one reported the point biserial correlation coefficient. The point-biserial correlation reflects the extent to which performance on an individual item aligns with overall test performance, thereby indicating the item’s ability to discriminate between high- and low-performing students [29]. Items with low or negative point-biserial values suggest poor discrimination and may require revision or removal. Similarly, the KR-20 if item deleted statistic shows how the overall internal consistency of the test would change if a particular item were removed [30]. If the reliability increases when an item is deleted, it suggests that the item is not functioning well and is lowering the overall test quality. Hence, along with the other indices, these should also be reported for a better presentation of the individual study and evidence synthesis.

Despite providing valuable insights into the performance of MCQs in Indian medical education, this study has several limitations. The included studies varied widely in sample size, subject area, and the number of MCQs analyzed, contributing to high heterogeneity across difficulty, discrimination, and distractor efficiency. Most studies were conducted in select states and institutions, limiting the generalizability of the findings to all regions or types of medical colleges in India. Differences in curriculum, teaching quality, and assessment practices were not fully accounted for.

Future original studies should adopt a multi-institutional collaborative design, collecting MCQ data prospectively from government, private, and institutions of national importance (INIs) across India. Establishing a centralized database over one to two years that includes item-level statistics from different medical subjects would provide a large and representative dataset. Such coordinated efforts would enable real-time monitoring of item quality, allow benchmarking of institutional performance against national standards, and facilitate longitudinal tracking of assessment quality. This type of collaborative original research would strengthen the evidence base, complement meta-analytic findings, and support the development of a national framework for high-quality medical assessments.

## Conclusions

This study provides a comprehensive synthesis of the performance of MCQs in undergraduate Indian medical education, highlighting overall moderate difficulty, generally acceptable discrimination, and considerable variability in distractor quality. The findings underscore the importance of routine psychometric evaluation to enhance the reliability, validity, and fairness of assessments. By providing empirical evidence on actual student performance, the study offers a foundation for establishing normative benchmarks, guiding assessment design, and informing faculty development programs. These insights have practical implications for educators, institutions, and policymakers, supporting data-driven strategies to improve assessment quality and promote consistency across diverse educational settings in India.
